# Supplementary material for: Characterisation of Phage Susceptibility Variation in Salmonella enterica Serovar Typhimurium DT104 and DT104b
Source: Microorganisms. 2021 Apr 17;9(4):865. doi: 10.3390/microorganisms9040865 (PMC8073726; doi:10.3390/microorganisms9040865)
Supplement: Supplementary file 1 [file microorganisms-09-00865-s001.zip › microorganisms-1156758-suppl Figure S1, Table S1,S2.pdf]

Supplementary Table S1. WGS-based identification of antimicrobial resistance determinants.

Strain ID	Phage Type	Resistance determinants	
DP_F10	DT104b	*aac(6')-Iaa*<br>*aadA2b*<br>*sul1*<br>*tet(G)*<br>*floR*<br>*blaCARB-2*	Amikacin, Tobramycin<br>Streptomycin<br>Sulfamethoxazole<br>Tetracycline<br>Chloramphenicol<br>Ampicillin
DP_N16	DT104b	*aac(6')-Iaa*<br>*aadA2b, aph(6)-Id, aph(3'')-Ib*<br>*sul1, sul2*<br>*tet(G)*<br>*floR*<br>*blaCARB-2*<br>*dfrA14*	Amikacin, Tobramycin<br>Streptomycin<br>Sulfamethoxazole<br>Tetracycline<br>Chloramphenicol<br>Ampicillin<br>Trimethoprim
DP_N28	DT104b	*aac(6')-Iaa*<br>*aadA2b, aph(6)-Id, aph(3'')-Ib*<br>*sul1, sul2*<br>*tet(G)*<br>*floR*<br>*blaCARB-2*<br>*dfrA14*	Amikacin, Tobramycin<br>Streptomycin<br>sulfamethoxazole<br>Tetracycline<br>Chloramphenicol<br>Ampicillin<br>Trimethoprim
JE_2727	DT104b	*aac(6')-Iaa*<br>*aadA2b*<br>*sul1*<br>*tet(G)*<br>*floR*<br>*blaCARB-2*	Amikacin, Tobramycin<br>Streptomycin<br>Sulfamethoxazole<br>Tetracycline<br>Chloramphenicol<br>Ampicillin
DT104b ref.	DT104b	*aac(6')-Iaa*	Amikacin, Tobramycin
JM_04.26	DT104	*sul1*<br>*aadA2b*<br>*aac(6')-Iaa*	Sulfamethoxazole<br>Streptomycin<br>Amikacin, Tobramycin
MC_04-0529	DT104	*aadA2b*<br>*aac(6')-Iaa*<br>*sul1*<br>*tet(G)*<br>*floR*<br>*blaCARB-2*	Streptomycin<br>Amikacin, Tobramycin<br>Sulfamethoxazole<br>Tetracycline<br>Chloramphenicol<br>Ampicillin
R13	DT104	*aac(6')-Iaa*<br>*aadA2b*<br>*sul1*<br>*tet(G)*<br>*floR*<br>*blaCARB-2*	Amikacin, Tobramycin<br>Streptomycin<br>Sulfamethoxazole<br>Tetracycline<br>Chloramphenicol<br>Ampicillin
TM75-404	DT104	*aac(6')-Iaa*	Amikacin, Tobramycin
DT104 ref.	DT104	*aadA2b*	Streptomycin
		aac(6')-Iaa	Amikacin, Tobramycin
		sul1	Sulfamethoxazole
		tet(G)	Tetracycline
		floR	Chloramphenicol
		blaCARB-2	Ampicillin
LT2	DT4	*aac(6')-Iaa*	Amikacin, Tobramycin

Supplementary Table S2. Distribution of potential genes coding for phage receptors among all strains of DT104 and DT104b

Gene	DP_F10 (DT104b)	DP_N16 (DT104b)	DP_N28 (DT104b)	JE_2727 (DT104b)	DT104b ref. (DT104b)	JM_04.26 (DT104)	MC_04-0529 (DT104)	R13 (DT104)	TM75-404 (DT104)	DT104 ref. (DT104)
Lipopolysaccharide biosynthesis protein WzxC	+	+	+	+	+	+	+	+	+	+
LPS O-antigen biosynthesis protein RfbU	+	+	-	+	+	+	+	+	+	+
Lipopolysaccharide N-acetylmannosaminouronosyltransferase (EC 2.4.1.180)	+	+	+	+	+	+	+	+	+	+
Lipopolysaccharide biosynthesis protein WzzE	+	+	+	+	+	+	+	+	+	+
Lipopolysaccharide export system protein LptC	+	+	+	+	+	+	+	+	+	+
Lipopolysaccharide export system protein LptA	+	+	+	+	+	+	+	+	+	+
Lipopolysaccharide ABC transporter, ATP-binding protein LptB	+	+	+	+	+	+	+	+	+	+
Lipopolysaccharide export system permease protein LptG	+	+	+	+	+	+	+	+	+	+
Lipopolysaccharide export system permease protein LptF	+	+	+	+	+	+	+	+	+	+
Lipopolysaccharide 1,2-N-acetylglucosaminetransferase (EC 2.4.1.56)	+	+	+	+	+	+	+	+	+	+
Lipopolysaccharide assembly protein LapB	+	+	+	+	+	+	+	+	+	+
Lipopolysaccharide core heptose(II)-phosphate phosphatase	+	+	+	+	+	+	+	+	+	+
Lipopolysaccharide core heptosyltransferase III	+	+	+	+	+	+	+	+	+	+
UDP-glucose:(heptosyl) LPS alpha1,3-glucosyltransferase WaaG	+	+	+	+	+	+	+	+	+	+
Lipopolysaccharide core heptose(I) kinase RfaP	+	+	+	+	+	+	+	+	+	+
Lipopolysaccharide 1,3-galactosyltransferase (EC 2.4.1.44)	+	+	+	+	+	+	+	+	+	+
Lipopolysaccharide 1,6-galactosyltransferase (EC 2.4.1.-)	+	+	+	+	+	+	+	+	+	+
UDP-glucose:(glucosyl)lipopolysaccharide alpha-1,2-glucosyltransferase (EC 2.4.1.58)	+	+	+	+	+	+	+	+	+	+
Lipopolysaccharide core heptose(II) kinase RfaY	+	+	+	+	+	+	+	+	+	+
Lipopolysaccharide core biosynthesis protein RfaZ	+	+	+	+	+	+	+	+	+	+
Lipopolysaccharide core heptosyltransferase I	+	+	+	+	+	+	+	+	+	+
Phosphoethanolamine transferase EptC [E.coli], specific for LPS heptose I residue	+	+	+	+	+	+	+	+	+	+
YihE protein, a ser/thr kinase implicated in LPS synthesis and Cpx signalling	+	+	+	+	+	+	+	+	+	+

Supplementary Figure S1. (A) ML phylogenetic tree of *Salmonella* Typhimurium DT104 and DT104b strains based on SNPs of prophags Salmon_118970_sal3; (B) ML phylogenetic tree of *Salmonella* Typhimurium DT104 and DT104b strains based on SNPs of prophage Salmon_ST64B; (C) ML phylogenetic tree of *Salmonella* Typhimurium DT104 and DT104b strains based on SNPs of prophage Gifsy-2; (D) ML phylogenetic tree of *Salmonella* Typhimurium DT104 and DT104b strains based on SNPs of prophage Gifsy-1.

A

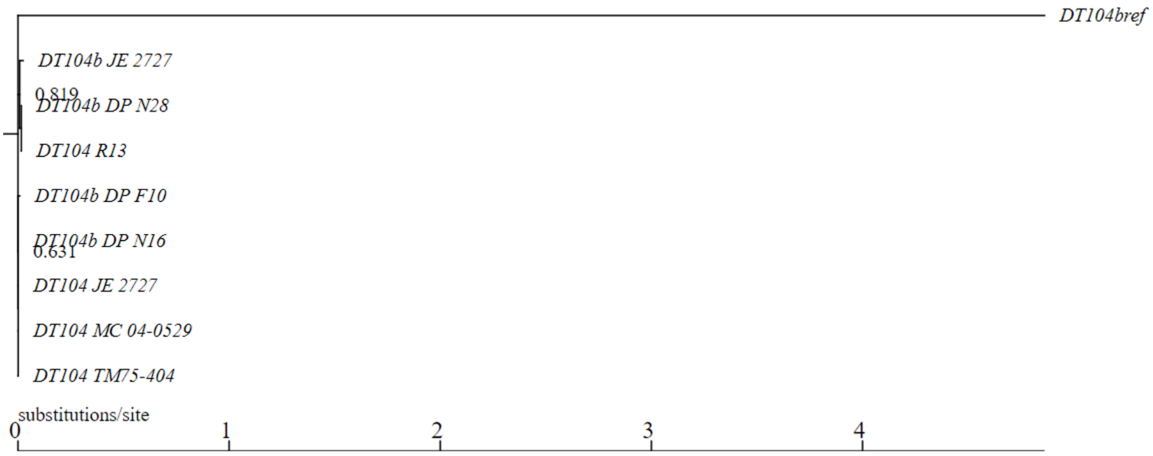

B

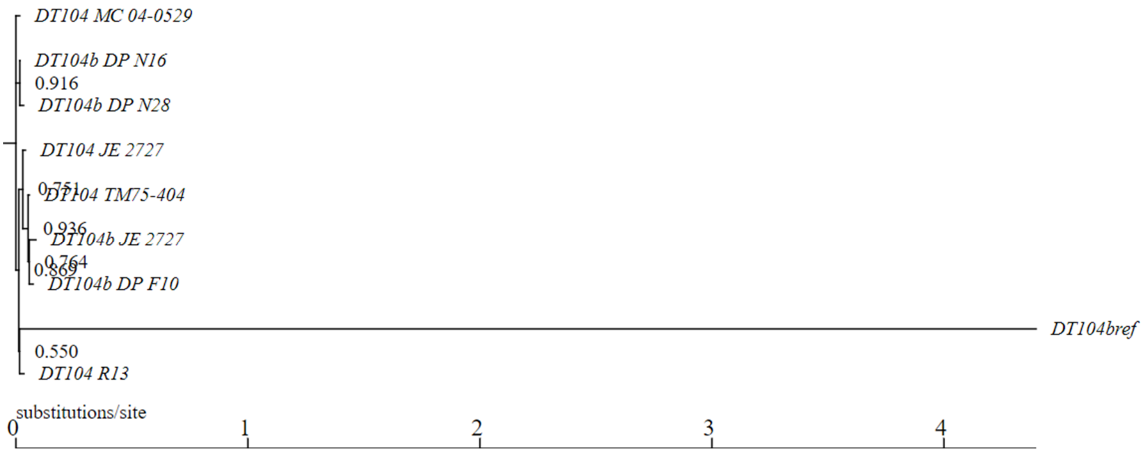

C

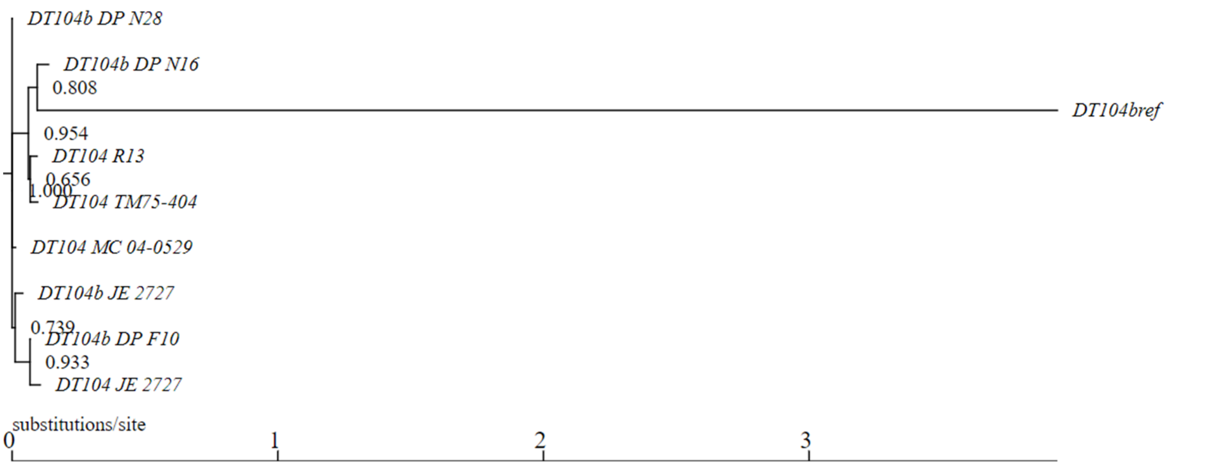

D

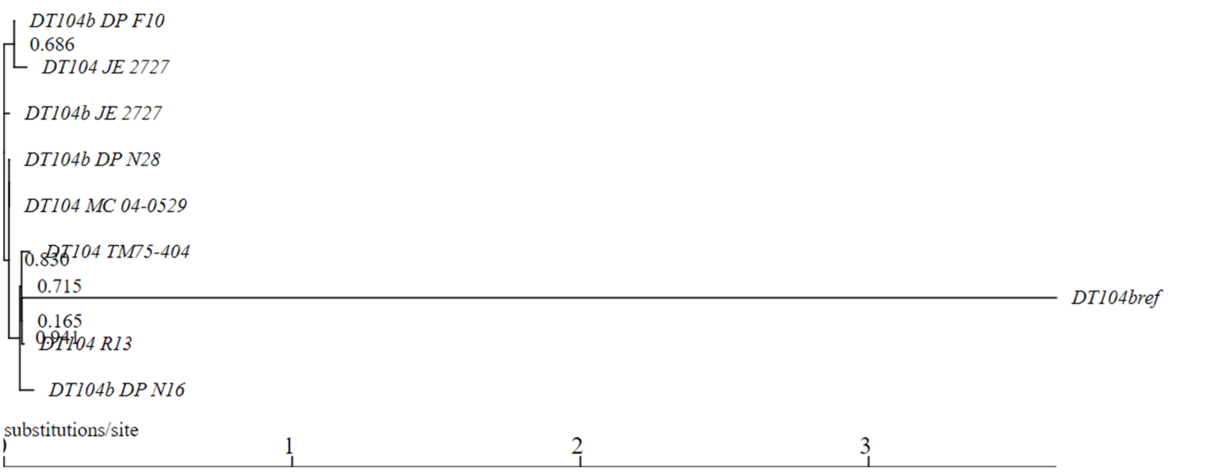
DT104b DP F10
0.686
DT104 JE 2727
DT104b JE 2727
DT104b DP N28
DT104 MC 04-0529
DT104 TM75-404
0.830
0.715
DT104bref
0.165
0.941
DT104 R13
DT104b DP N16
substitutions/site
0
1
2
3
